# Prevalence of sarcopenic obesity in the older non-hospitalized population: a systematic review and meta-analysis

**DOI:** 10.1186/s12877-024-04952-z

**Published:** 2024-04-22

**Authors:** Yuhong Luo, Yanqiu Wang, Shuao Tang, Ludan Xu, Xinyu Zhao, Mengya Han, Yuhua Liu, Yan Xu, Binru Han

**Affiliations:** 1 School of Nursing, Capital Medical University, Beijing, China; 2 Xuanwu Hospital, Capital Medical University, No 45., Changchun Street, Xicheng District, 100055 Beijing, China

**Keywords:** Older adults, Sarcopenic obesity, Prevalence, Meta-analysis, Systematic review

## Abstract

**Background:**

Sarcopenic obesity emerges as a risk factor for adverse clinical outcomes in non-hospitalized older adults, including physical disabilities, metabolic diseases, and even mortality. In this systematic review and meta-analysis, we investigated the overall SO prevalence in non-hospitalized adults aged ≥ 65 years and assessed the sociodemographic, clinicobiological, and lifestyle factors related to SO.

**Methods:**

We searched the PubMed, Embase, Cochrane Library, and Web of Science databases for studies reporting the prevalence of SO from database inception to October 2023. Two researchers independently screened the literature, evaluated the study quality, and extracted the data. Both fixed- and random-effects models were used in the meta-analysis to estimate the pooled SO prevalence and perform subgroup analyses. Publication and sensitivity bias analyses were performed to test the robustness of the associations.

**Results:**

Among 46 studies eligible for review and a total of 71,757 non-hospitalized older adults, the combined prevalence of SO was 14% (95% CI:11–17%, I^2^ = 99.5%, *P* < 0.01). Subgroup analysis according to lifestyle factors demonstrated that the SO prevalence was 17% (95% CI: 8–29%, I^2^ = 99.5%, *P* < 0.01) in older adults without exercise habits. Regarding clinicobiological factors, older adults with a history of falls (15% [95% CI: 10–22%, I^2^ = 82%, *P* < 0.01]), two or more chronic diseases (19% [95% CI: 10–29%, I^2^ = 97%, *P* < 0.01]), functional impairment (33% [95% CI: 29–37%, I^2^ = 0%, *P* = 0.95]), cognitive impairment (35% [95% CI: 9–65%, I^2^ = 83%, *P* = 0.02]), osteoporosis (20% [95% CI: 8–35%, I^2^ = 96%, *P* < 0.01]), high fasting glucose level (17% [95% CI: 1–49%, I^2^ = 98%, *P* < 0.01]), or the use of antipsychotics (13% [95% CI: 2–28%, I^2^ = 0%, *P* = 0.32]) exhibited a higher SO prevalence.

**Conclusion:**

SO prevalence is high among non-hospitalized older adults, especially those with functional and cognitive impairments. Thus, SO is a potential problem for the aging population; implementation of planned interventions in the community is needed to reduce the prevalence and adverse outcomes of SO.

**Supplementary Information:**

The online version contains supplementary material available at 10.1186/s12877-024-04952-z.

## Background

The coexistence of sarcopenia and increased fat mass is referred to as sarcopenic obesity (SO) [1]. Compared with sarcopenia or obesity alone, the synergistic effect of muscle loss and obesity leads to a higher risk of adverse outcomes such as falls, physical disabilities, and fractures and is closely related to the occurrence of metabolic diseases such as cardiovascular disease, diabetes, and non-alcoholic fatty liver disease, as well as death [2–4]. SO is an important health problem, and its prevalence and mortality are increasing worldwide, especially in the older adult population [5]. Apart from its impact on health status, SO also has considerable independent effects on healthcare expenses [6–8].

By recognizing the prevalence and risk factors for SO, healthcare professionals and primary care clinicians can provide early detection, diagnosis, and intervention for patients who potentially have SO [9–10]. However, the prevalence of SO is not yet clearly established and can vary by as much as 26, mainly because of the use of different evaluation methods, criteria, and cutoff points applied to determine muscle mass and fat mass [11]. The diagnostic criteria for SO currently used in research are based on the coexistence of sarcopenia and obesity, leading to the application of a diverse diagnostic criteria for sarcopenia and obesity [12]. A Korean cohort study using muscle mass and waist circumference to diagnose SO demonstrated an SO prevalence of 41.6%, with 35.2% prevalence in men and 48.2% in women [13]. A cross-sectional study in China defined sarcopenia as low muscle mass and strength and obesity as high body fat percentage; they showed an SO prevalence of only 4.0%, including 7.0% in men and 2.4% in women [14].

Furthermore, the prevalence rate of SO also differs when a different diagnostic criteria was adopted for the same population. A Brazilian longitudinal study included older adults aged > 65 years and found that the prevalence of SO using two diagnostic criteria for SO (muscle mass combined with body fat percentage vs. muscle mass and strength combined with body fat percentage) was 29.3% and 19.3%, respectively [7, 15]. In addition, the prevalence of SO is also affected by clinicobiological and lifestyle factors in older adults; older adults with chronic conditions were 1.8 times more likely to develop SO than the general population [16]. A study by Son et al. found that active physical activity may be negatively associated with the development of sarcopenia and sarcopenic obesity in older adults [17]. Based on the above research results, we hypothesized that there are differences in sociodemographic, clinicobiological, and lifestyle factors among older adults that would impact the corresponding SO prevalence.

Therefore, we conducted a systematic review and meta-analysis to investigate the overall prevalence of SO in non-hospitalized adults aged ≥ 65 years and examine the association of various health-related factors with the disease. Our results can provide information to ensure better allocation of healthcare resources and early healthcare decision making for older patients with SO.

## Methods

### Literature search

The Preferred Reporting Items for Systematic Reviews and Meta-Analyses (PRISMA) statement guidelines were followed for the calculation and reporting of meta-analysis data [18]. Literature searches were conducted using the EMBASE, PubMed, Web of Science, and Cochrane Library databases; the search period was from database inception through October 2023. The following search terms were used: “Sarcopenia,” “Obesity,” “Sarcopenic Obesity,” “Aged,” and “Elderly.” The references identified in the relevant publications were also reviewed to identify additional studies. The detailed search strategy used for each database is presented in Additional File S1.

### Inclusion and exclusion criteria

Studies that met the following criteria were included: (1) participants: aged ≥ 65 years in nursing homes or communities, without sex, race, or regional restrictions; (2) exposure: SO (the patients should have both sarcopenia and obesity, and the diagnostic criteria and cutoff values for sarcopenia and obesity were not restricted); (3) outcome: SO prevalence (if there were any additional data required to confirm, we contacted the corresponding author of the study twice within a 1-month period); (4) study design: cohort studies and cross-sectional studies (baseline data were extracted from cohort studies); (5) there were no limitations on the language of publication, year of publication, or publication status. The study exclusion criteria were as follows: (1) studies that did not provide a clear diagnostic criterion of SO; (2) studies including participants with specific diseases; (3) reviews, lectures, case reports, or articles in which the data were evidently abnormal or missing (and the author could not be contacted).

### Study selection and data extraction

The identified studies were stored in a reference management software (EndNote, Clarivate, Philadelphia, PA, United States). Literature screening and data extraction were independently performed by two reviewers. If the included articles were not written in English or Chinese, the study team made a preliminary translation of the included documents with the help of translation software and invited translators to proofread and revise them before the study team performed the reading and data extraction. Any disagreements between the reviewers were resolved by discussion with a third reviewer. We extracted the first author’s name, year of publication, study name, country in which the study was conducted, sample size, diagnostic criteria of sarcopenia and obesity, body mass index (BMI) and other study parameters, and the prevalence of SO.

### Quality assessment and publication bias

Two researchers independently evaluated the risk of bias in the included studies using the Joanna Briggs Institute’s Critical Appraisal Checklist for Prevalence Studies [19]. There were 9 items in total: (1) “Was the sample frame appropriate to address the target population?;” (2) “Were study participants sampled in an appropriate way?;” (3) “Was the sample size adequate?;” (4) “Were the study participants and setting described in detail?;” (5) “Was the data analysis conducted with sufficient coverage of the identified sample?;” (6) “Were valid methods used for identification of the condition?:” (7) “Was the condition measured in a standard, reliable way for all of the participants?;” (8) “Was there an appropriate statistical analysis?;” and (9) “Was the response rate adequate?.” For each item, the study was assigned a “yes,” “no,” “unclear,” or “not applicable” remark. The total number of “yes” answers was counted per study, with a greater number of “yes” answers indicating a higher quality of the study. Studies were eligible if more than five “yes” answers were achieved [5]. Any disagreements were resolved by discussion or through consultation with a third senior researcher. Publication bias was tested using Egger’s funnel plots.

### Statistical analysis

We used the R software (version 4.3.2, R Foundation for Statistical Computing, Vienna, Austria) for all statistical analyses. The combined prevalence and 95% confidence interval (95% CI) of SO in adults aged ≥ 65 years were calculated. Heterogeneity among the studies was assessed using Q and I^2^ statistic indices. A significant Q value (*P* < 0.1) indicated a lack of homogeneity among the studies; I^2^ = 0 indicated that the inconsistency among the results is not statistically different (I^2^ < 50% indicated low inconsistency, I^2^ ≥ 50% indicated high inconsistency). If the heterogeneity test results were *P* > 0.1 and I^2^ < 50%, the homogeneity of the study was considered good, and a fixed-effects model was adopted; otherwise, the random-effects model was adopted.

Subgroup analyses were performed based on the diagnostic criteria of SO, study design, geographical region, age, sex, race, education level, physical activity, fall history, number of chronic diseases, comorbidities, high fasting glucose level, and drug use.

## Results

### Characteristics of the included studies

**Fig. 1:**
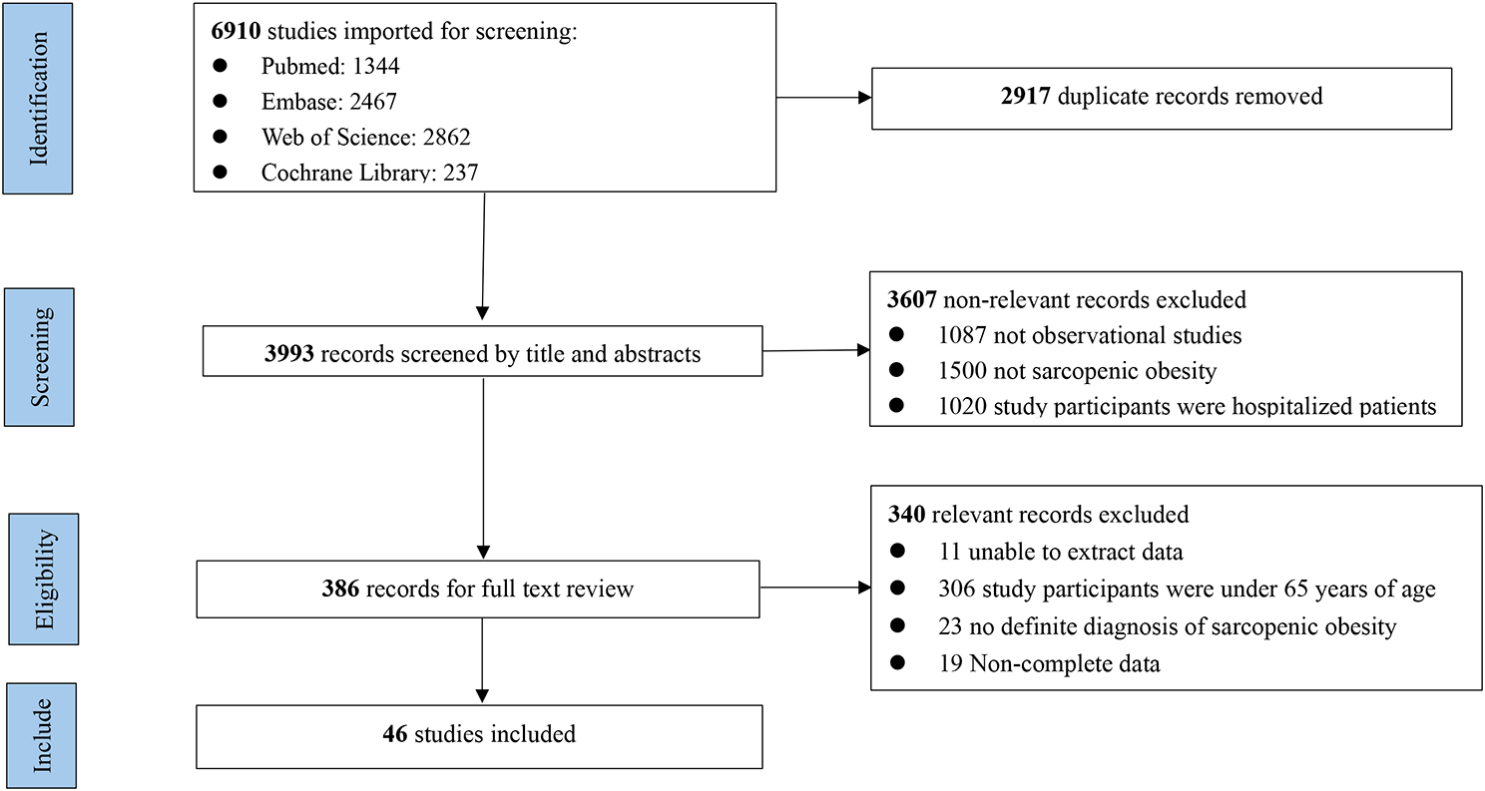
Flow diagram of search

A flow chart of the study selection process and exclusion criteria is shown in Fig. 1. According to the search criteria, 6,910 articles were found during the literature search; after excluding duplicate references, 3,993 remained for further screening. We filtered the results by title, abstract, and full text. Finally, 46 studies met the inclusion and exclusion criteria. Among them, 17 [6, 9, 13, 15, 20–32] were cohort studies, and 29 [7–8, 10, 14, 17, 33–56] were cross-sectional studies. Articles published in the last 3 years (post-2020) accounted for 28 [6–7, 9–10, 15, 20–21, 23, 26–31, 36, 39, 41, 44–47, 49–50, 52–56] studies. The total number of participants included in this review was 71,757 from studies with sample sizes ranging from 64 [34] to 7,852 [6]. Seven [31, 34–35, 37–39, 50] studies included only women, three [21, 24–25] included only men, and the remaining studies included participants of both sexes. The included studies used varying diagnostic criteria for sarcopenia (low muscle mass, low muscle strength, or low muscle strength and mass) combined with different obesity criteria (percentage of body fat [PBF], BMI, or waist circumference [WC]) to diagnose SO. Among them, 14 [6–8, 10, 13, 17, 20, 22, 28, 34, 45, 47, 51, 54] studies used low muscle mass as the diagnostic criterion for sarcopenia, 10 [9, 20, 26, 29, 40–41, 49–50, 55–56] studies used low muscle strength as the diagnostic criterion for sarcopenia, and 20 [14–15, 21–25, 27, 30–31, 35–36, 39, 42–44, 46, 48, 52–53] studies used low muscle mass plus low muscle strength as the diagnostic criterion for sarcopenia. In addition, one [6] study, a multicenter population study, did not provide a specific cutoff for their obesity diagnostic criteria (PBF). Two [37–38] studies used the appendicular fat-free mass (calculated as follows: -14.529 + [17.989 × height in meters] + [0.1307 × total fat mass in kg]) truncation value as the diagnostic criterion for SO and did not use the diagnostic method of sarcopenia combined with obesity. Detailed characteristics of the included studies are displayed in Additional File S2.

### Study quality evaluation

**Fig. 2:**
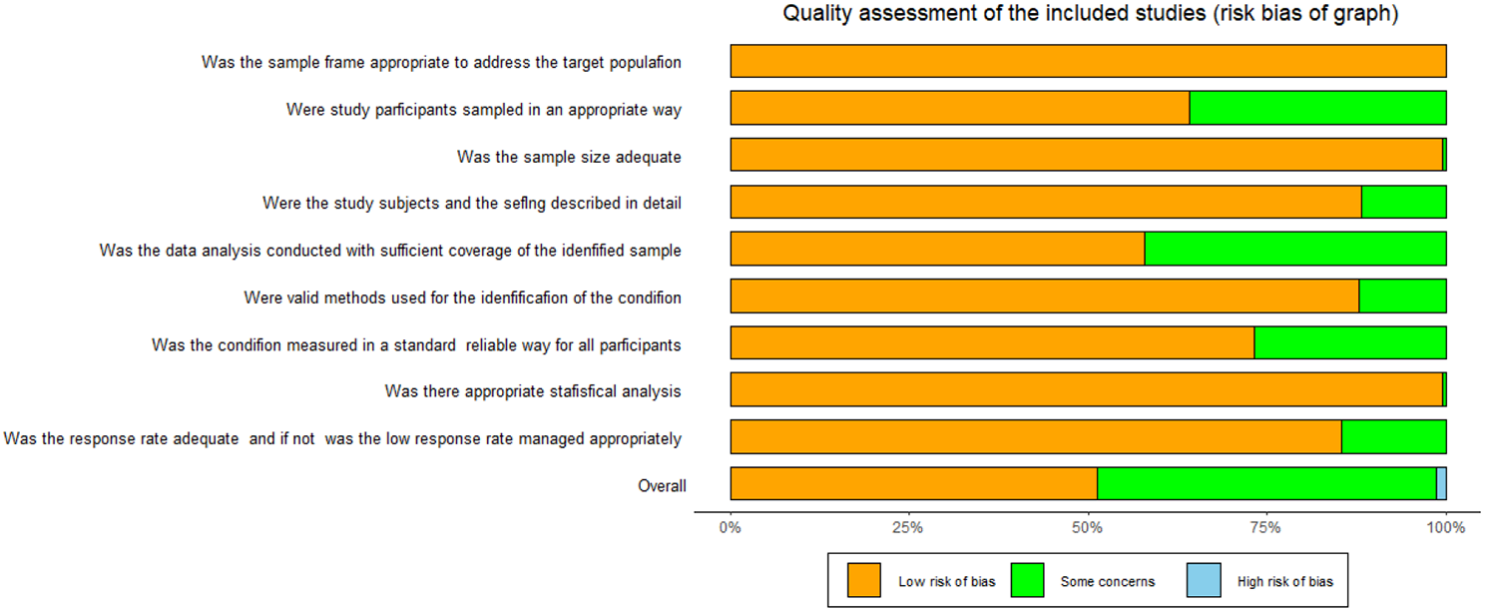
Quality assessment of the included studies (risk bias of graph). *Notes* Judgments about each risk of bias item are presented as percentages across all included studies

Most studies were of fair [6, 8, 10, 2–13, 16, 19, 25–26, 29, 31, 32, 35–37, 42, 48, 51–53] or high [7, 9, 15, 21–25, 28–29, 31, 33, 35, 36, 40–43, 45, 49, 51, 52–56] quality because they scored “yes” for at least five items in the quality assessment checklist. Specifically, 26 [7, 9, 15, 21–25, 28–29, 31, 33, 35–36, 40–43, 45–49, 51–52, 56] studies scored “yes” for 8–9 items, 18 [6, 8, 13–14, 17, 20, 26–27, 30, 32, 34, 37, 39, 44, 50, 53–55] studies scored “yes” for 6–7 items, and 2 [38, 10] studies scored “yes” for 5 items. Detailed assessment results for the included studies are displayed in Fig. 2 and Additional File S3.

### Overall prevalence of SO in non-hospitalized older adults ≥ 65 years

The prevalence of SO in the 46 included studies varied from 3% [40] to 55% [6]. The pooled SO prevalence was 14% (95% CI: 11–17%), with a high level of heterogeneity (I^2^ = 99.5%, *P* < 0.01). Therefore, we used a random-effects model (Additional File S4).

### Subgroup analysis of diagnostic criteria, study design, sociodemographic, lifestyle and clinicobiological factors

#### Diagnostic criteria of SO

We found a higher prevalence of SO when muscle mass alone was used to diagnose sarcopenia compared with that when muscle strength alone or muscle mass plus muscle strength was used. The pooled prevalence of SO diagnosed using low muscle mass combined with different diagnostic criteria for obesity was 21% (95% CI: 13–29%, I^2^ = 99.5%, *P* < 0.01, 14 studies [6–8, 10, 13, 17, 22, 28, 33–34, 45, 47, 51, 54]). Among them, the pooled prevalence of SO diagnosed using low muscle mass + BMI, low muscle mass + PBF, and low muscle mass + WC was 18% (95% CI: 9–29%, I^2^ = 99%, *P* < 0.01, 2 studies [33, 45]), 29% (95% CI: 13–49%, I^2^ = 98%, *P* < 0.01, 5 studies [6, 15, 34, 51–52]), 18% (95% CI: 7–32%, I^2^ = 98%, *P* < 0.01, 5 studies [8, 13, 17, 47, 54]), respectively (Table 1).

**Table 1:** Subgroup analysis of diagnostic criteria, study design, sociodemographically, lifestyle and clinicobiological

Subgroup	Meta-analysis					Heterogeneity		Effect model
	Number of included studies ( n)	Number of patients ( n)	Cases of SO( n)	Prevalence (95% Cl)		P	I^2^ (%)	
**Diagnostic criteria of sarcopenia**								
Low muscle mass	14	32,213	8265	21%(13%, 29%)		*P* < 0.01	99.5%	Random
Low muscle strength	10	19,096	2691	12%(8%, 16%)		*P* < 0.01	98%	Random
Low muscle mass + Low muscle strength	20	20,169	2039	10%(6%, 14%)		*P* < 0.01	98%	Random
**Diagnostic criteria of SO**								
Low muscle mass + BMI	2	7311	1312	18%(9%, 29%)		*P* < 0.01	99%	Random
Low muscle mass + PBF%	5	10,760	4700	29%(13%, 49%)		*P* < 0.01	98%	Random
Low muscle mass + WC	5	13,056	2096	18%(7%, 32%)		*P* < 0.01	98%	Random
Low muscle strength + BMI	5	4397	584	11%(5%, 19%)		*P* < 0.01	98%	Random
Low muscle strength + PBF%	2	1308	87	8%(2%, 19%)		*P* < 0.01	96%	Random
Low muscle mass + Low muscle strength + PBF%	9	11,191	1027	10%(6%, 14%)		*P* < 0.01	98%	Random
Low muscle mass + Low muscle strength + BMI	4	4071	679	18%(15%, 20%)		*P* = 0.07	57%	Random
Low muscle mass + Low muscle strength + BMI + WC + PBF%	3	2005	144	9%(3%, 17%)		*P* < 0.01	94%	Random
**Study design**								
Cohort study	17	30,836	3742	13%(9%, 17%)		*P* < 0.01	99%	Random
Cross-sectional study	29	40,921	5523	13%(10%, 16%)		*P* < 0.01	99%	Random
**Geographical region**								
Asia	22	43,722	6109	12%(9%, 16%)		*P* < 0.01	99%	Random
Europe	10	7930	989	11%(7%, 15%)		*P* < 0.01	97%	Random
Eurasian	2	1180	187	14%(3%, 31%)		*P* < 0.01	98%	Random
South America	6	954	224	22%(12%, 35%)		*P* < 0.01	90%	Random
North America	2	7422	1068	16%(10%, 24%)		*P* < 0.01	98%	Random
Oceania	2	2647	216	8%(5%, 11%)		*P* < 0.01	88%	Random
**Age**								
65–74	9	12,860	1700	13%(9%, 18%)		*P* < 0.01	98%	Random
75–84	7	5597	1006	17%(11%, 25%)		*P* < 0.01	97%	Random
≥ 85	4	1166	162	23%(11%, 37%)		*P* < 0.01	92%	Random
**Gender**								
Female	38	34,477	7072	15%(11%, 19%)		*P* < 0.01	99.2%	Random
Male	34	28,414	4506	13%(10%, 17%)		*P* < 0.01	99%	Random
**Race**								
White	3	4494	586	23%(10%, 38%)		*P* < 0.01	97%	Random
Black	4	1519	233	17%(12%, 23%)		*P* = 0.02	71%	Random
**Education level**								
< High school	7	14,398	3100	16%(9%, 24%)		*P* < 0.01	99%	Random
High school to some college	6	6191	923	14%(6%, 245)		*P* < 0.01	98%	Random
College or more	8	4031	446	11%(5%, 19%)		*P* < 0.01	96%	Random
**Physical activity**								
Moderate physical activity	13	14,450	3599	15%(8%, 23%)		*P* < 0.01	99.5%	Random
Vigorous physical activity	4	1362	173	12%(4%, 23%)		*P* < 0.01	98%	Random
Not very/not at all	7	7904	1908	17%(8%, 29%)		*P* < 0.01	99.5%	Random
**Fall history**								
Yes	5	867	136	15%(10%, 22%)		*P* < 0.01	82%	Random
No	5	5159	547	9%(4%, 14%)		*P* < 0.01	98%	Random
**Number of chronic diseases**								
0	3	6794	1493	16%(3%, 37%)		*P* < 0.01	99.5%	Random
1	4	5784	431	10%(2%, 23%)		*P* < 0.01	99%	Random
≥ 2	4	2549	548	19%(10%, 29%)		*P* < 0.01	97%	Random
**Comorbidities**								
Cancer	2	1762	200	8%(2%, 18%)		*P* < 0.01	95%	Random
Lung disease	5	1878	354	16%(9%, 25%)		*P* < 0.01	85%	Random
Hypertension	10	10,356	1349	12%(8%, 17%)		*P* < 0.01	98%	Random
Dyslipidemia	6	4140	758	15%(6%, 27%)		*P* < 0.01	99%	Random
Functional disabilities	2	675	239	33%(29%, 37%)		*P* = 0.95	0%	Fixed
Osteoporosis	4	1892	488	20%(8%, 35%)		*P* < 0.01	96%	Random
Arthritis	6	5334	1027	17%(10%, 25%)		*P* < 0.01	95%	Random
Probable Dementia	2	72	29	35%(9%,65%)		*P* = 0.02	83%	Random
Cerebrovascular disease	5	820	106	9%(5%, 14%)		*P* < 0.01	71%	Random
Diabetes	14	4497	825	14%(10%, 19%)		*P* < 0.01	95%	Random
Heart disease	10	3523	592	14%(9%, 19%)		*P* < 0.01	92%	Random
Depressive Symptoms	4	837	168	16%(7%, 28%)		*P* < 0.01	96%	Random
**High fasting glucose**								
Yes	2	1207	105	17%(1%, 49%)		*P* < 0.01	98%	Random
No	2	1754	89	12%(1%, 42%)		*P* < 0.01	98%	Random
**Drug use**								
Oral hypoglycemic agents	2	392	46	11%(6%, 17%)		*P* = 0.09	66%	Random
Use of anti-psychotics	2	30	4	13%(2%, 28%)		*P* = 0.32	0%	Fixed
Statin use	3	1028	60	6%(4%, 7%)		*P* = 0.53	0%	Fixed

*Abbreviations* SO: sarcopenic obesity; BMI: body mass index; PBF: body fat percentage; WC: waist circumference

The pooled prevalence of SO diagnosed using low muscle strength combined with different diagnostic criteria for obesity was 12% (95% CI: 8–16%, I^2^ = 98%, *P* < 0.01, 10 studies [9, 20, 26, 29, 40–41, 49, 50, 55–56]). Among them, the pooled prevalence of SO diagnosed using low muscle strength + BMI was 11% (95% CI: 5–19%, I^2^ = 98%, *P* < 0.01, 5 studies [9, 29, 40, 50, 55]), whereas that diagnosed using low muscle strength + PBF was 8% (95% CI: 2–19%, I^2^ = 96%, *P* < 0.01, 2 studies [41, 49]) (Table 1).

The pooled prevalence of SO diagnosed using low muscle mass + low muscle strength combined with different diagnostic criteria for obesity was 10% (95% CI: 6–14%, I^2^ = 98%, *P* < 0.01, 20 studies [14–15, 21–25, 27, 30–31, 35–36, 39, 42–44, 46, 48, 52–53]). Among them, the pooled prevalence of SO diagnosed using low muscle mass + low muscle + PBF, low muscle mass + low muscle + BMI, and low muscle mass + low muscle + BMI + WC + PBF was 10% (95% CI: 6–14%, I^2^ = 98%, *P* < 0.01, 9 studies [14–15, 22, 24, 35, 42–43, 46, 52]), 18% (95% CI: 15–20%, I^2^ = 57%, *P* = 0.07, 4 studies [15, 31, 44, 48]), and 9% (95% CI: 9–17%, I^2^ = 94%, *P* < 0.01, 3 studies [27, 36, 53]), respectively (Table 1).

#### Study design

Our findings suggest that the study design (cross-sectional/cohort study) had no effect on the prevalence of SO. The pooled SO prevalence for cohort and cross-sectional studies was 13% (95% CI: 9–17%, I^2^ = 99%, *P* < 0.01) and 13% (95% CI: 10–16%, I^2^ = 99%, *P* < 0.01), respectively (Table 1).

#### Geographical region

The prevalence of SO is higher in South and North America than that in Asia, Europe, and Oceania. The pooled prevalence of SO in Asia, Europe, Eurasia, South America, North America, and Oceania was 12% (95% CI: 9–16%, I^2^ = 99%, *P* < 0.01, 22 studies [8, 10, 13–14, 17, 22, 26–27, 29, 33, 41–47, 51–54, 56]), 11% (95% CI: 7–15%, I^2^ = 97%, *P* < 0.01, 10 studies [23, 25, 28, 30, 31, 32–35, 36, 39–48, 49 ]), 14% (95% CI: 3–31%, I^2^ = 98%, *P* < 0.01, 2 studies [40, 55]), 22% (95% CI: 12–35%, I^2^ = 90%, *P* < 0.01, 6 studies [7, 15, 34, 37–38, 50]), 16% (95% CI: 10–24%, I^2^ = 98%, *P* < 0.01, 2 studies [9, 19]), and 8% (95% CI: 5–11%, I^2^ = 88%, *P* < 0.01, 2 studies [21, 24]), respectively (Table 1).

#### Age, sex, race, and education level

Subgroup analyses based on sociodemographic variables of the included study population revealed higher SO prevalence rates among those aged ≥ 85 years, females, Whites, and those with a high school or less than high school education. The pooled prevalence of SO in individuals aged 65–74, 75–84, and ≥ 85 years was 13% (95% CI: 9–18%, I^2^ = 98%, *P* < 0.01, 9 studies [6–7, 9, 15, 20, 23, 32, 46–47]), 17% (95% CI: 11–25%, I^2^ = 97%, *P* < 0.01, 7 studies [6–7, 9, 20, 23, 46–47]), and 23% (95% CI: 11–37%, I^2^ = 92%, *P* < 0.01, 4 studies [7, 9, 20, 23]), respectively (Table 1).

The pooled prevalence of SO in females and males was 15% (95% CI: 11–19%, I^2^ = 99.2%, *P* < 0.01, 38 studies [9–10, 13–15, 22, 23–26, 43–45, 52–56]) and 13% (95% CI: 10–17%, I^2^ = 99%, *P* < 0.01, 34 studies [6–10, 13–15, 20–30, 32–33, 36, 40–43, 45–49, 51–52, 56]), respectively (Table 1).

The pooled prevalence of SO in Whites and Blacks was 23% (95% CI: 10–38%, I^2^ = 97%, *P* < 0.01, 3 studies [7, 15, 20]) and 17% (95% CI: 12–23%, I^2^ = 71%, *P* = 0.02, 4 studies [7, 15, 20, 31]), respectively (Table 1).

The pooled prevalence of SO in individuals with educational levels < high school, high school to some college, and college or more was 16% (95% CI: 9–24%, I^2^ = 99%, *P* < 0.01, 7 studies [6–8, 20, 42, 47, 56]), 14% (95% CI: 6–24%, I^2^ = 98%, *P* < 0.01, 6 studies [6–8, 20, 42, 47]), and 11% (95% CI: 5–19%, I^2^ = 96%, *P* < 0.01, 8 studies [6–8, 19–21, 42, 47, 51]), respectively (Table 1).

#### Physical activity

Among older adults, those who do not exercise regularly tend to have a higher prevalence of SO compared with those who maintain a consistent exercise routine. The pooled prevalence of SO in individuals whose physical activity levels were moderate, vigorous, and not much/not at all was 15% (95% CI: 8–23%, I^2^ = 99.5%, *P* < 0.01, 13 studies [6–8, 15, 17, 20, 26, 45–47, 51, 54, 56]), 12% (95% CI: 4–23%, I^2^ = 98%, *P* < 0.01, 4 studies [8, 17, 46–47]), and 17% (95% CI: 8–29%, I^2^ = 99.5%, *P* < 0.01, 7 studies [6, 10, 14, 44–45, 49, 52]), respectively (Table 1).

#### Fall history

Older adults with a history of falls were more likely to experience SO than those without such history. The pooled prevalence of SO in older adults with and without a history of falls was 15% (95% CI: 10–22%, I^2^ = 82%, *P* < 0.01, 5 studies [9, 21, 27, 46, 51]) and 9% (95% CI: 9–14%, I^2^ = 98%, *P* < 0.01, 5 studies [9, 21, 27, 46, 51]), respectively (Table 1).

#### Number of chronic diseases

Subgroup analyses showed that older adults with no chronic disease or only one chronic disease had lower SO prevalence than those with multiple chronic diseases. The pooled prevalence of SO for individuals with 0, 1, and ≥ 2 chronic diseases was 16% (95% CI: 3–37%, I^2^ = 99.5%, *P* < 0.01, 3 studies [6, 45, 52]), 10% (95% CI: 2–23%, I^2^ = 99%, *P* < 0.01, 4 studies [6, 8, 45, 52]), and 19% (95% CI: 10–29%, I^2^ = 97%, *P* < 0.01, 4 studies [6, 8, 45, 52]), respectively (Table 1).

#### Comorbidities

Subgroup analysis based on disease type demonstrated higher SO prevalence among older adults with functional or cognitive impairment and osteoporosis. The pooled prevalence of SO in individuals with cancer, lung disease, hypertension, dyslipidemia, functional disabilities, osteoporosis, arthritis, probable dementia, cerebrovascular disease, diabetes, heart disease, and depressive symptoms was 8% (95% CI: 2–18%, I^2^ = 95%, *P* < 0.01, 2 studies [20, 42]), 16% (95% CI: 9–25%, I^2^ = 85%, *P* < 0.01, 5 studies [9, 20, 23, 26, 39]), 12% (95% CI: 8–17%, I^2^ = 98%, *P* < 0.01, 10 studies [8–9, 15, 20, 22, 27, 29, 41, 47, 51]), 15% (95% CI: 6–27%, I^2^ = 99%, *P* < 0.01, 6 studies [8, 29, 33, 41, 47, 51]), 33% (95% CI: 29–37%, I^2^ = 0%, *P* = 0.95, 2 studies [34, 37]), 20% (95% CI: 8–35%, I^2^ = 96%, *P* < 0.01, 4 studies [8–9, 25–26]), 17% (95% CI: 10–25%, I^2^ = 95%, *P* < 0.01, 6 studies [7, 9, 19, 25–26, 49]), 35% (95% CI: 9–65%, I^2^ = 83%, *P* = 0.02, 2 studies [9, 22], 9% (95% CI: 5–14%, I^2^ = 71%, *P* < 0.01, 5 studies [19, 22, 28, 40, 49]), 14% (95% CI: 10–19%, I^2^ = 95%, *P* < 0.01, 14 studies [7–9, 14, 19, 22–23, 26, 28, 39–40, 45, 49, 53]), 14% (95% CI: 9–19%, I^2^ = 92%, *P* < 0.01, 10 studies [9, 14, 19, 22, 25, 28, 37, 39–40, 49]), and 16% (95% CI: 7–28%, I^2^ = 96%, *P* < 0.01, 4 studies [9, 22, 28, 40]), respectively (Table 1).

#### High fasting glucose levels

Higher fasting glucose levels in older adults were associated with a higher incidence of SO. The pooled prevalence of SO in individuals with and without high fasting glucose levels was 17% (95% CI: 1–49%, I^2^ = 98%, *P* < 0.01, 2 studies [7, 45]) and 12% (95% CI: 1–42%, I^2^ = 98%, *P* < 0.01, 2 studies [7, 45]), respectively (Table 1).

#### Drug use

We also analyzed the impact of medication usage on the prevalence of SO among older adults and found that those using antipsychotics had a higher rate of SO occurrence. The pooled prevalence of SO in individuals using oral hypoglycemic agents, anti-psychotics, and statins was 11% (95% CI: 6–17%, I^2^ = 66%, *P* = 0.09, 2 studies [23, 39]), 13% (95% CI: 2–28%, I^2^ = 0%, *P* = 0.32, 2 studies [26, 40]), and 6% (95% CI: 4–7%, I^2^ = 0%, *P* = 0.53, 3 studies [23, 40, 49]), respectively (Table 1).

Table 1 Subgroup analysis of diagnostic criteria, study design, sociodemographic, lifestyle and clinicobiological factors.

## Publication bias

**Fig. 3:**
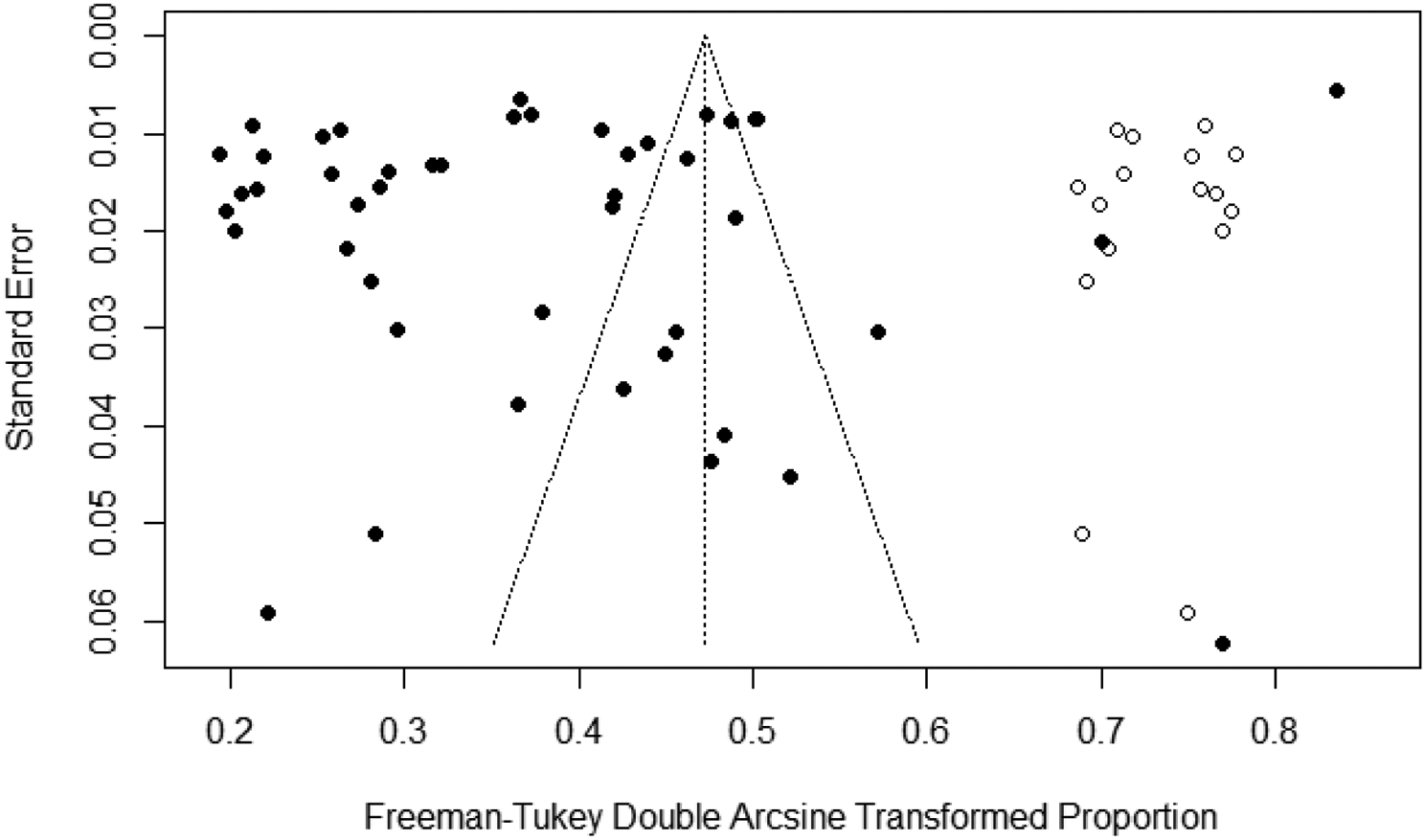
Egger’s funnel plots for testing publication bias

A funnel plot was created to represent the total prevalence of SO; the plot showed an asymmetric distribution of the study points. Egger’s test results (*P* = 0.05) also suggested the possibility of a publication bias. A nonparametric shear complement method was used to estimate the number of missing studies and evaluate the influence of publication bias on the results, which showed significant differences in the results before and after splicing. The prevalence of SO, calculated before and after trimming, was 14% (95% CI: 11–17%) and 20% (95% CI: 16–24%), respectively, suggesting that publication bias had a great influence on the stability of the results (Fig. 3).

## Sensitivity analysis

To assess the stability of the results, we performed a sensitivity analysis on the 46 included studies by sequentially excluding individual studies. After arbitrarily excluding one study, the combined conversion rate based on the random-effects model was 14% (95% CI: 11–17%), indicating that it had little influence on the combined effect size. Therefore, the results of our meta-analysis are stable and reliable (Additional File S5).

## Discussion

We assessed the overall prevalence of SO in non-hospitalized adults aged ≥ 65 years and comprehensively assessed the sociodemographic, clinicobiological, and lifestyle factors associated with SO. The global prevalence of SO was 14%, higher than the 11% prevalence reported by Gao et al. [5], which may be due to the older age of our study population. Moreover, the prevalence of SO was higher when muscle mass measurements alone were used to diagnose sarcopenia compared with when muscle strength alone (12%) or muscle mass plus muscle strength (10%) was used for diagnosis. Study design (cross-sectional study/cohort study) had no effect on the prevalence of SO. Furthermore, the prevalence of SO in South (22%) and North America (16%) was higher than that in Asia (12%), Europe (11%), and Oceania (8%), which may be because the South and North American studies included in this study mainly used muscle mass to diagnose sarcopenia and the higher rates of obesity in North America and South America [55].Various factors, including comorbidities, resulted in the SO prevalence varying broadly from 6–35%.

Consistent with multiple study findings [20, 27, 34, 37], our review shows that the prevalence of SO is influenced by its diagnostic criteria. When muscle mass alone was used to diagnose sarcopenia, the prevalence of SO was higher than that when muscle strength alone or the combined diagnosis of muscle mass and strength was used. The prevalence of SO was the lowest when diagnosed using muscle strength combined with PBF and highest when diagnosed using muscle mass combined with PBF. Different diagnostic criteria for sarcopenia have been developed for different ethnic groups [56–59]. These diagnostic criteria comprehensively evaluate muscle mass, strength, and function to diagnose sarcopenia and suggest a joint definition of sarcopenia using appendicular skeletal muscle mass and one or two functional parameters (grip strength and/or gait speed). However, there are still some studies that diagnose sarcopenia solely based on muscle mass [6, 27, 43, 45, 52]. The application of consistent definitions would improve the comparability of sarcopenia prevalence in future cohort studies.

Obesity is typically diagnosed using BMI, WC, or PBF; the prevalence of obesity as defined using BMI is lower than that defined using WC or waist-to-hip ratio [60]. Fat accumulation and redistribution associated with muscle loss do not necessarily increase BMI [61]. Therefore, when BMI is used for diagnosis, the prevalence of obesity is relatively low, and missed diagnosis may occur. WC (abdominal fat accumulation), the only measure of body fat distribution independently associated with impaired mobility, had a better correlation with SO [62]. Additionally, follicle-stimulating hormone levels increase along with the rapid decline of estrogen in postmenopausal females, leading to increased visceral fat accumulation [63]. This transfer of fat deposits to the center of the body can increase WC in females [64]. Therefore, the WC method is more sensitive to the diagnosis of obesity in females than in males. When obesity was defined by PBF, the prevalence of SO also increased with age [24, 65]. This explains the major age-related changes in body composition, including increased body fat and sarcopenia. Finally, regardless of the diagnostic method for SO, low muscle mass, low muscle strength, and obesity are significant risk factors for disability and increased mortality [14, 20, 22]. Therefore, prevention of sarcopenia and obesity in the relatively healthy older adult population should be a major goal.

The prevalence of SO varies demographically. In this study, the prevalence in individuals aged 75–84 years was 17%; in those ≥ 85 years, it was as high as 23%. Aging increases body fat and insulin resistance, leading to chronic diseases as well as decreased muscle strength and mass [66]. Therefore, advanced age is associated with a higher prevalence of SO. There was no significant sex difference in the prevalence of SO, potentially because male muscle mass gradually decreases with age, whereas female muscle mass does not decrease significantly with age [67]. However, in early menopause, female muscle mass and function decline significantly owing to a significant decrease in estrogen [68]. Additionally, our results showed that the prevalence of SO was higher in Whites than in Blacks. Different cultural backgrounds, dietary patterns, and physical activity levels may contribute to racial differences in the prevalence of SO. Moreover, lower education levels were associated with a higher SO prevalence. Education level is a predictor of employment type and health behavior. Therefore, providing health education regarding SO to older people of lower socioeconomic status is essential

 Regarding lifestyle, consistent with findings from multiple studies [8, 45, 49], older adults who were moderately and intensely active had a lower prevalence of SO than those who were inactive. Park et al. [45] demonstrated that all types of exercises were beneficial to SO and observed that moderate-to-high intensity exercise was highly correlated with skeletal muscle index and grip strength. A meta-analysis of randomized controlled trials showed that exercise, particularly resistance exercise, is critical for improving body composition and physical performance in patients with muscle-reducing obesity [69]. Another study showed that post-exercise macronutrient supplementation (equivalent to 200 kcal) during home-based interval walking training enhanced skeletal muscle mass and strength compared with exercise alone [70]. Calorie restriction combined with moderate aerobic exercise was shown to reduce muscle mass loss in older adults with obesity [71]. These findings support the importance of exercise in the intervention of sarcopenic obesity. Future studies are needed to longitudinally compare the combined effects of nutrition and exercise interventions in sarcopenia and SO.

 We found that the prevalence of SO in older adults with a history of falls was 15%. Depending on the number of chronic diseases and medication usage, the prevalence of SO is between 6% and 35%. Fall history was independently related to balance confidence and fall risk [33]. Balance confidence and fear of falling lead to further self-restriction and avoidance of activities of daily living, with reduced physical activity, whereas sedentary behavior is associated with obesity and low muscle strength in older adults. Additionally, falls also cause dysfunction in older adults, and dysfunction is a risk factor for SO [25, 28]. Correspondingly, we found that the prevalence of SO among disabled older adults was high at 33%. The main reason may be related to low physical activity due to physical limitations. A prospective study of 1,851 Japanese residents aged 65 years and older showed that, in addition to aging, major factors associated with sarcopenia were depressed mood and cognitive impairment [72]. We found that the prevalence of SO in older adults with depression and those with cognitive impairment was 16% and 35%, respectively. Correspondingly, the prevalence of SO in older adults using antipsychotic drugs was higher than that in those taking oral hypoglycemic drugs and statins. Similarly, in obese women, increased grip strength is associated with a reduced cognitive impairment risk [73]. The synergistic effect between cognitive impairment and SO is unclear. Chronic inflammation is a basic common pathology of dementia, obesity, and sarcopenia [28, 74–75]. Patients with SO may be in a chronic inflammatory state, leading to a strong correlation between SO and dementia. Additionally, because cognitive and motor performance depend on the nervous system, nervous system damage may lead to both cognitive and motor function decline [76].

Further prospective and interventional studies are needed to clarify the causal relationship between SO prevalence and cognitive impairment. We found that the prevalence of SO was as high as 20% in the older adult population with osteoporosis. This may be related to the loss of muscle mass caused by osteoporosis. Additionally, women with SO are more likely to have high blood sugar, whereas men with SO are more likely to have osteoporosis and dyslipidemia [13]. Further research is needed to establish the causal pathways and identify mediators of the association, particularly modifiable factors, to prevent comorbidities and sarcopenic obesity. Additionally, men and women with SO-related adverse outcomes should be addressed differently

Our study had certain strengths. First, our study highlights the particular importance of sociodemographic, clinicobiological, and lifestyle factors in the prevalence of SO among non-hospitalized older adults. Second, we conducted an extensive literature search and included high-quality studies that produced reliable results. Nevertheless, our study also had some limitations. First, heterogeneity remained high after subgroup analysis; in addition to racial and ethnic differences, different definitions or diagnostic parameters may also contribute to heterogeneity. Several examples for the varying definitions were those for the criteria (gait speed and/or grip strength) and calculation of skeletal muscle mass index (ASMM/height^2^ or ASMM/BMI) and cutoff points between the morphometric and functional criteria (grip strength 30 kg–26 kg; gait speed 0.8 m/s or 1.0 m/s) [56–59]. However, heterogeneity is often unavoidable in meta-analyses of observational studies and does not necessarily invalidate meta-analysis results [77]. Second, nutritional status and nutrient intake are closely related to the metabolism of muscle and fat; however, because the nutrition-related data reported in studies included in our review were mostly continuous data, a single rate meta-analysis was not possible. Therefore, the relationship between nutritional status and nutrient intake and the prevalence of SO needs further exploration

## Conclusions

 The higher prevalence of SO in non-hospitalized older adults, especially among those with dysfunction and cognitive impairment, is a potential problem for the aging population. Our findings provide valuable information to clinicians who plan community interventions, as they can address these risk factors and thus reduce the prevalence of SO. Owing to the diversity of SO diagnostic criteria and demarcation points, the comparability of data is limited. Therefore, our findings provide a useful basis for future researchers to work from as they build a consensus on the diagnosis of SO.

### Electronic supplementary material

Below is the link to the electronic supplementary material.

Supplementary Material 1

Supplementary Material 2

Supplementary Material 3

Supplementary Material 4

Supplementary Material 5

## Data Availability

All extracted data used in this review has been reported in the text, fgures, tables, and Additional file.

## Abbreviations

BMI: body mass index
95% CI: 95% confidence interval
PBF: percentage of body fat
PRISMA: Preferred Reporting Items for Systematic Reviews and Meta-Analyses
SO: sarcopenic obesity
WC: waist circumference
