# Supplementary material for: Prevalence of sarcopenic obesity in the older non-hospitalized population: a systematic review and meta-analysis
Source: BMC Geriatr. 2024 Apr 22;24:357. doi: 10.1186/s12877-024-04952-z (PMC11036751; doi:10.1186/s12877-024-04952-z)
Supplement: Supplementary file 1 — Supplementary Material 1 [file 12877_2024_4952_MOESM1_ESM.docx]

**Additional file 1.** Search strategies for each database

| **Number** | **Databases** | **Search terms** |
| --- | --- | --- |
| 1 | PubMed | #1 "Sarcopenia"[MeSH Terms]  #2 Sarcopenia [Title/Abstract]  #3 #1 OR #2  #4 "Obesity"[MeSH Terms]  #5 Obesity [Title/Abstract]  #6 #4 OR #5  #7 Sarcopenic Obesity [Title/Abstract]  #8 #3 AND #6  #9 #7 OR #8  #10 "Aged"[MeSH Terms]  #11 "Elderly"[Title/Abstract] OR "Aged"[Title/Abstract]  #12 #10 OR #11  #13 #9 AND #12 |
| 2 | Web of Science | #1 Sarcopenia (Topic) and Obesity (Topic)  #2 Sarcopenic Obesity (Topic)  #3 #1 OR #2  #4 Aged (Topic)  #5 #3 AND #4 |
| 3 | Embase | #1 'sarcopenia'/exp  #2 sarcopenia:ti,ab,kw  #3 #1 OR #2  #4 'obesity'/exp  #5 obesity:ti,ab,kw  #6 #4 OR #5  #7 'sarcopenic obesity':ti,ab,kw  #8 'aged'/exp  #9 aged:ti,ab,kw OR elderly:ti,ab,kw  #10 #8 OR #9  #11 #3 AND #6  #12 #7 OR #11  #13 #10 AND #12 |
| 4 | Cochrane Library | #1 MeSH descriptor: [Aged] in all MeSH products  #2 (Aged):ti,ab,kw OR (Elderly):ti,ab,kw  #3 #1 OR #2  #4 MeSH descriptor: [Sarcopenia] explode all trees  #5 (Sarcopenia):ti,ab,kw  #6 #4 OR #5  #7 (obesity):ti,ab,kw  #8 MeSH descriptor: [Obesity] explode all trees  #9 #7 OR #8  #10 (Sarcopenic Obesity):ti,ab,kw  #11 #6 AND #9  #12 #11 OR #10  #13 #12 AND #3 |
