# Supplementary material for: Prevalence of sarcopenic obesity in the older non-hospitalized population: a systematic review and meta-analysis
Source: BMC Geriatr. 2024 Apr 22;24:357. doi: 10.1186/s12877-024-04952-z (PMC11036751; doi:10.1186/s12877-024-04952-z)
Supplement: Supplementary file 2 — Supplementary Material 2 [file 12877_2024_4952_MOESM2_ESM.docx]

**Additional file 2.** Descriptive characteristics of the studies included in this review

| First Author, Year | Country | Study design | Sample (N) Total-Male-Female | SO, (%) Total-  Male-Female | Mean Age | BMI (kg/m²) | Grip strength（kg） | Fat (%) | Sarcopenia definition | Obesity definition |
| --- | --- | --- | --- | --- | --- | --- | --- | --- | --- | --- |
| Nascimento 2018 [34] | Brazil | Cross-sectional | 64-0-64 | 31-0-31 | 68.19±5.06 | 31.19±4.35 | NA | 45.17±4.46 | ALM/BMI＜0.512 | PBF ≥ 38% |
| Aibar-Almazán 2018 [35] | Spanish | Cross-sectional | 235-0-235 | 44-0-44 | 69.21±7.56 | 29.78±4.20 | 18.49 ± 5.12 | NA | SMI/h^2^＜6.42kg/m^2^;  HGS＜20kg;  GS≤0.8m/s | PBF ≥ 35% |
| Baek 2013[33] | Korea | Cross-sectional | 3483-1466-2017 | 805-318-435 | NA | 27.1±0.1 | NA | 29.1±0.20 | ASM/Wt < 32.5% for men and 25.7% for women | BMI ≥ 25kg/m^2^ |
| Batsis 2021[20] | America | Cohort | 5822-2578-3244 | 750-318-432 | NA | 34.2±4.2 | 20.2±7.4 | NA | HGS < 35.5 kg for men and < 20 kg for women; HGS/BMI < 1.05 for men and < 0.79 for women | BMI ≥ 30kg/m^2^ or WC ≥ 88cm for women; BMI ≥ 30kg/m^2^ or WC ≥ 102 for men |
| Campos 2021[15] | Brazil | Cohort | 270-81-189 | 52-27-25 | 77.5±5.92 | 28.8±9.2 | 20.3±6.9 | 37.8±7.8 | HGS < 16kg and ALML < 6.0kg/m^2^ for women; HGS < 27kg and ALML < 7.0kg/m^2^ for men | PBF≥38% for women and ≥27% for men |
| Daskalopoulou 2020[6] | Cuba, Dominican Republic, Peru, Mexico, Puerto Rico, China | Cohort | 7852-2806-5046 | 4231-1452-2518 | NA | NA | NA | NA | SMI cut-offs for women ranged from 0.515 to 0.572 and for men ranged from 0.882 to 0.964 | NA |
| Scott 2023[21] | Australia | Cohort | 1416-1416-0 | 136-136-0 | 76.5±5.2 | 31.1 ± 4.4 | 30.1 ± 7.0 | 35.8 ± 4.1 | HGS < 27kg or 5CST < 15s and ALM/h < 7.0kg/m^2^ | BMI≥30 kg/m^2^ or WC≥90cm |
| De Campos 2020[7] | Brazil | Cross-sectional | 270-81-189 | 79-51-27 | 77.5±5.92 | NA | NA | 35.6±4.5 | ALMI < 6.0 kg/m^2^ for women and < 7.0kg/m^2^ for men; HGS < 16kg for women and < 27kg for men | PBF≥38% for women and ≥27% for men |
| Diago-Galmés 2021[36] | Spain | Cross-sectional | 190-35-155 | 32-7-25 | NA | NA | 14.1 ± 1.3 | NA | HGS < 16kg for women and < 27kg for men; ASM < 15kg for women and < 20kg for men; GS≤0.8m/s | BMI≥30kg/m^2^; WC < 102cm for men and > 88cm for women; PBF ≥ 38% |
| Dondero 2022[9] | America | Cohort | 1600-739-861 | 318-149-169 | NA | NA | NA | NA | HGS < 27kg men and < 16 kg for women; SPPB ≤ 8 | BMI ≥ 30kg/m^2^ |
| dos Santos 2014[37] | Brazil | Cross-sectional | 149-0-149 | 32-0-32 | 67.2±6.1 | 26.98 ±4.41 | NA | 42.22±6.52 | AFFM/h^2^ ≤ 5.45 kg/m^2^ | NA |
| Du 2019[14] | China | Cross-sectional | 631-213-418 | 25-15-10 | NA | 23.9 ± 1.7 | 18.6 ± 5.5 | 43.2 ± 5.0 | ASMI < 5.24kg/m^2^ for women and < 6.66 kg/m^2^ for men; HGS < 15kg for women and < 24kg for men; GS < 0.8m/s | PBF% > 35.9% for women and > 27.2 for men |
| Dutra 2017[38] | Brazil | Cross-sectional | 130-0-130 | 27-0-27 | 66.7±5.2 | NA | NA | 47.0 ± 6.8 | AFFM/m^2^≤3.4 kg/m^2^ | NA |
| Kim 2013[22] | Korea | Cohort | 2031-1032-999 | 368-225-143 | 75.9±3.9 | 24.1±2.8 | NA | 31.5±7.9 | ASM/h^2^ < 7.00 kg/m^2^ for men and < 5.40 kg/m^2^ for women; HGS < 28 kg for men and < 18 kg for women; GS< 1.0 m/s | PBF% > 38.8% for women and > 28.2 for men |
| Fonfría-Vivas 2023[39] | Spain | Cross-sectional | 95-0-95 | 7-0-7 | 76.0±5.7 | 30.55±3.79 | NA | NA | HGS < 16kg or 5CST > 15s and ASM/h^2^ <5.5 kg/m^2^ | BMI ≥ 30kg/m^2^ or WC ≥ 88 cm |
| Ulugerger 2023[23] | Ireland | Cohort | 175-55-120 | 22-3-19 | 33.5±3.2 | 14.3±4.3 | NA | NA | SMI/h^2^ < 9.2kg/m^2^ for women and < 7.4kg/h^2^ for men; HGS < 16kg for women and < 27kg for men; GS < 0.8m/s | BMI ≥ 30.0 kg/m^2^ |
| Halil 2014[40] | Turkey | Cross-sectional | 711-357-352 | 157-49-106 | 78.5±7.4 | NA | NA | NA | CC < 31cm; HGS ≤21kg for women and ≤32kg for men | BMI ≥ 30.0kg/m^2^ |
| Scott 2018[24] | Australia | Cohort | 1231-1231-0 | 80-80-0 | 80.3 ± 6.5 | 27.2 ± 2.3 | 27.4 ± 6.9 | 34.8 ± 3.5 | ALM/h^2^ < 7.26 kg/m^2^; HGS < 30kg; GS ≤ 0.8m/s | PBF% ≥ 30% |
| Hwang 2023[10] | Korea | Cross-sectional | 812-325-487 | 134-53-81 | 77.8 ± 1.8 | 28.1 ± 2.5 | NA | NA | SMI < 0.789 for men and < 0.521 for women | BMI ≥ 25kg/m^2^; WC > 90cm for men and > 80cm for women |
| Ida 2023[41] | Japan | Cross-sectional | 310-191-119 | 42-25-17 | 75.3±7.3 | NA | NA | NA | HGS ＜ 28kg for men and ＜ 18kg for women; 5CST ≥ 12s | PBF% ≥ 29% for men and ≥ 41% for women |
| Shinya 2016[42] | Japan | Cross-sectional | 1731-875-856 | 64-32-32 | 77.1 ±5.2 | NA | NA | NA | SMI/h^2^ < 7.0kg/m^2^ for men and < 5.8kg/m^2^ for women; HGS <3 0kg for men and <20kg for women; GS<1.26m/s | PBF% ≥ 29.7% for men and ≥ 37.2% for women |
| Kemmler 2017[25] | Germany | Cohort | 965-965-0 | 40-40-0 | 77.3 ± 4.9 | NA | 36.2 ± 7.3 | 30.2 ± 6.5 | SMI/h^2^ < 7.18kg/m^2^; GS < 0.8m/s; HGS<30kg | BMI≥30kg/m^2^ |
| Kera 2017[43] | Japan | Cross-sectional | 1283-483-800 | 105-27-78 | 73.6 ± 5.4 | 23.5 ± 2.8 | NA | 31.5 ± 7.2 | SMI/h^2^ < 7.09kg/m^2^ for men and < 5.91kg/m^2^; HGS < 25kg for men and < 20kg for women; GS <1.0m/s | PBF% ≥ 26.9% for men and ≥33.6% for women |
| Kim 2022[44] | Korea | Cross-sectional | 932-319-613 | 155-NA-NA | 71.8±8.1 | 26.0 ± 0.1 | 15.6 ± 0.3 | 36.2 ± 0.3 | SMI/h^2^ < 7.0kg/m^2^ for men and <5.7kg/m^2^ for women; HGS < 28.0kg for men and < 18.0kg for women | BMI ≥ 25kg/m^2^ |
| Lee 2021[45] | Korea | Cross-sectional | 3828-1635-2193 | 507-172-335 | 73.1 ± 0.2 | 27.8 ± 0.2 | NA | NA | SMI < 0.789 for men and < 0.512 for women | BMI ≥ 25kg/m^2^ |
| Lim 2018[8] | Korea | Cross-sectional | 3492-1850-1642 | 812-316-496 | 69.7±8.0 | 26.7±3.9 | NA | NA | ASM/Wt < 32.5% for men and < 25.7% for women | WC > 90cm for men and > 85cm for women |
| Lim 2010[13] | Korea | Cohort | 565-287-278 | 235-101-134 | 73.9±7.7 | 26.6±2.9 | NA | NA | ASM/Wt < 29.9% for men and < 25.1% for women | WC > 90cm for men and > 85cm for women |
| Lu 2022[46] | China | Cross-sectional | 1047-581-826 | 140-81-59 | 71.91 ± 5.59 | 24.65 ± 3.32 | 28.01±9.8 | NA | ASMI ≤ 7.0kg/m^2^ for men and ≤ 5.7kg/m^2^ for women; HGS < 28kg for men and < 18kg for women; GS < 1.0m/s or 5CST≥12s | PBF% ≥ 25% for men and ≥ 35% for women |
| Morikawa 2023[26] | Japan | Cohort | 3882-1690-2192 | 807-277-530 | 74.6±5 | NA | NA | NA | HGS < 28kg for men and < 18kg for women; GS < 1.0m/s; 5CST ≥ 12s or SPPB ≤8 | PBF% ≥ 25% or visceral fat content ≥ 100cm^2^ |
| Park 2023[47] | Korea | Cross-sectional | 2971-1295-1696 | 132-62-70 | 74.0±0.5 | 24.1±0.2 | NA | 34.2±0.4 | ASMI ≤ 7.0kg/m^2^ for men and ≤ 5.4kg/m^2^ for women | WC ≥ 90cm for men and ≥ 85cm for women |
| Pedrero-Chamizo 2015[48] | Spain | Cross-sectional | 2747-645-2102 | 442-100-342 | 72.4 ± 5.4 | 68.3 ± 10.5 | NA | 39.2 ± 5.4 | ASMI ≤ 7.0kg/m^2^ for men and ≤ 5.7kg/m^2^ for women; HGS < 28kg for men and < 18kg for women; GS < 1.0m/s or 5CST ≥ 12s | BMI ≥ 25kg/m^2^ |
| Peng 2021[27] | China | Cohort | 765-325-440 | 29-7-22 | 80.35±8.13 | 24.71±2.44 | 14.47±5.16 | 38.68±5.29 | SMI < 0.789 for men and < 0.512 for women; HGS < 28kg for men and < 18kg for women; GS<1.0m/s | BMI ≥ 27kg/m^2^; WC > 90cm for men and > 80cm for women; PBF% > 25% for men and >30% for women |
| Andrea P Rossi 2020[28] | Italy | Cohort | 274-97-177 | 23-9-14 | 71.35 ± 2.35 | 26.55 ± 1.89 | NA | 42.03 ± 6.13 | ASMI ≤ 7.0kg/m^2^ for men and ≤ 5.18kg/m^2^ for women | FM% > 31.17% for men and > 44.01% for women |
| Schluessel 2023[49] | Germany | Cross-sectional | 998-500-498 | 45-25-20 | 75.6 ±6.5 | NA | 28.2 ±9.9 | 34.5 ±7.1 | HGS < 28kg for men and < 18kg for women; 5CST ≥ 12s | PBF% ≥ 29% for men and ≥ 41% for women |
| Someya 2022[29] | Japan | Cohort | 1615-684-931 | 76-35-41 | 73.1±5.4 | 26.9 ± 1.9 | 20.0 ± 5.3 | 27.3 ± 6.1 | HGS < 28kg for men and < 18kg for women; GS < 1.0m/s | BMI ≥ 25kg/m^2^ |
| Son 2019[17] | Korea | Cross-sectional | 3367-NA-NA | 738-NA-NA | 72.4 ± 0.34 | 27.3 ± 0.16 | NA | 39.8 ± 0.19 | ASM/Wt < 32.5% for men and < 25.7% for women | WC ≥ 90cm for men and ≥ 85cm for women |
| Teixeira 2023[50] | Brazil | Cross-sectional | 71-0-71 | 3-0-3 | NA | NA | NA | NA | HGS < 20kg | BMI ≥ 27kg/m^2^ |
| Von 2020[30] | Sweden | Cohort | 521-202-319 | 36-23-13 | 75.6 ± 0.3 | 25.7 ± 4.1 | 24.2 ± 4.3 | 39.7 ± 7.3 | 5CST ≥ 15s; HGS < 27kg for men and < 16kg for women; SMI/h^2^ < 5.75kg/m^2^ for women and < 8.5 kg/m^2^ for men | BMI ≥ 30kg/m^2^; PBF% ≥ 30% or WC>88cm |
| Yang 2015[51] | China | Cross-sectional | 844-448-396 | 61-33-28 | 76.5 ± 6.8 | 23.8±2.1 | NA | NA | SMI/h^2^ < 6.87kg/m^2^ for men and < 5.46kg/m^2^ | PBF% ≥ 27.82% for men and ≥ 37.61% for women |
| Yang 2022[52] | China | Cross-sectional | 2372-943-1429 | 149-81-68 | 67.6±5.9 | NA | NA | NA | SMI/h^2^ < 7kg/m^2^ for men and < 5.7kg/m^2^ for women; HGS < 28kg for men and ＜18kg for women; GS < 1.0m/s | PBF% ≥ 30% |
| Mo 2021[53] | China | Cross-sectional | 1050-347-703 | 83-NA-NA | 69.9±7.5 | NA | 13.7 ±0.5 | 24.7 ±3.7 | SMI/h^2^ < 7kg/m^2^ for men and < 5.7kg/m2; HGS < 28kg for men and < 18kg for women; GS<1.0m/s | BMI≥30kg/m^2^; WC ≥ 80cm for women and ≥ 90cm for men; PBF% ≥ 40% for women and ≥ 30% for men |
| Mendham 2021[31] | South African | Cohort | 122-0-122 | 30-0-30 | NA | 33.1±6.5 | 19.6 ± 4.5 | 48.6 ± 5.9 | HGS/BMI < 0.56 and SMI < 0.512 | BMI ≥ 30kg/m^2^ |
| Lee 2023[54] | Korea | Cross-sectional | 2661-NA-NA | 179-NA-NA | 72.93 ± 0.48 | 24.75 ± 0.17 | NA | NA | SMI/h^2^ < 7kg/m^2^ for men and < 5.4kg/m^2^ for women | WC ≥ 85cm for women and ≥ 90cm for men |
| Altinkaynak 2023[55] | Turkey | Cross-sectional | 397-202-195 | 30-NA-NA | 77.95±7.94 | 64.4 ± 16.9 | NA | NA | HGS < 27kg for men and < 16kg for women | BMI ≥ 30kg/m^2^ |
| Jang 2023[56] | Korea | Cross-sectional | 3690-1645-2045 | 463-107-356 | 73.2 ± 0.1 | 24.6 ± 0.1 | 19.5 ± 0.1 | NA | HGS < 28kg for men and < 18kg for women | WC ≥ 85cm for women and ≥ 90cm for men |
| Moreno-Franco 2018[32] | Spain | Cohort | 1730-830-935 | 298-147-151 | NA | NA | NA | NA | SMI/h^2^ ≤ 11.69kg/m^2^ for men and ≤9.19 kg/m^2^ for women | PBF% ≥ 42.7% for women and ≥ 32.69% for men |

Abbreviations: SO: sarcopenic obesity; ALM: appendicular lean mass; BMI: body mass index; PBF: body fat percentage; ASM: appendicular skeletal muscle mass; h: height; HGS: hand-grip strength; GS: gait speed; Wt: weight; BMI: body mass index; WC: waist circumference; ALMI: appendicular lean mass index; SMI: skeletal muscle index; 5CST: 5-time chair stand test; SPPB: short physical performance battery; AFFM: appendicular fat-free mass; ASMI: appendicular skeletal mass index; CC: calf circumference; FM: fat mass; NA: not reported
