# Supplementary figures and images for: Prevalence of sarcopenic obesity in the older non-hospitalized population: a systematic review and meta-analysis
Source: BMC Geriatr. 2024 Apr 22;24:357. doi: 10.1186/s12877-024-04952-z (PMC11036751; doi:10.1186/s12877-024-04952-z)

**Additional file 3.** Quality assessment of the included studies (risk bias of summary)


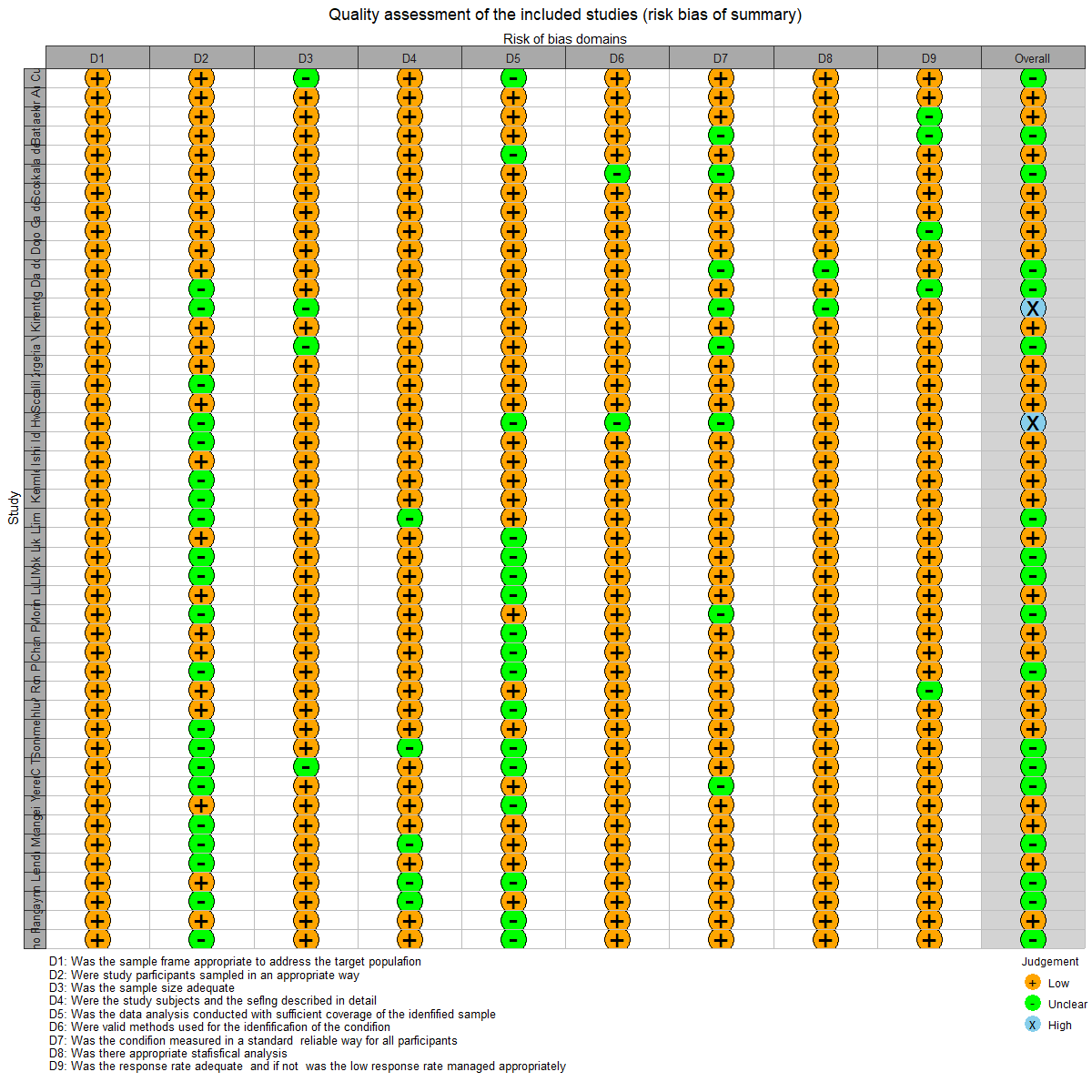

Supplement: Supplementary file 3 — Supplementary Material 3 [file 12877_2024_4952_MOESM3_ESM.docx]

**Additional file 4.** Forest plot of overall prevalence of SO in non-hospitalized elderly ≥65 years


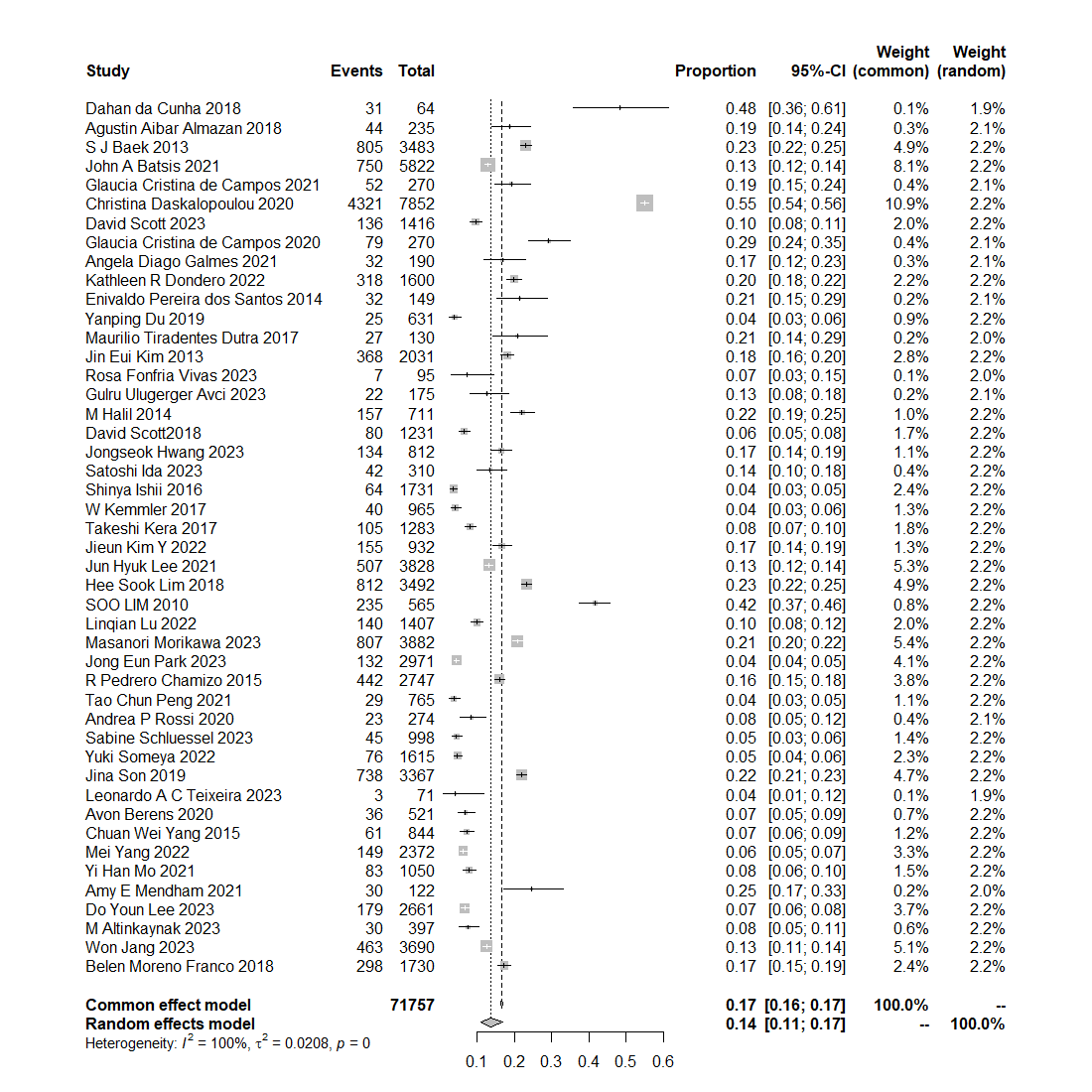

Supplement: Supplementary file 4 — Supplementary Material 4 [file 12877_2024_4952_MOESM4_ESM.docx]

**Additional file 5.** Sensitivity analysis of all included studies. CI: Confidence interval


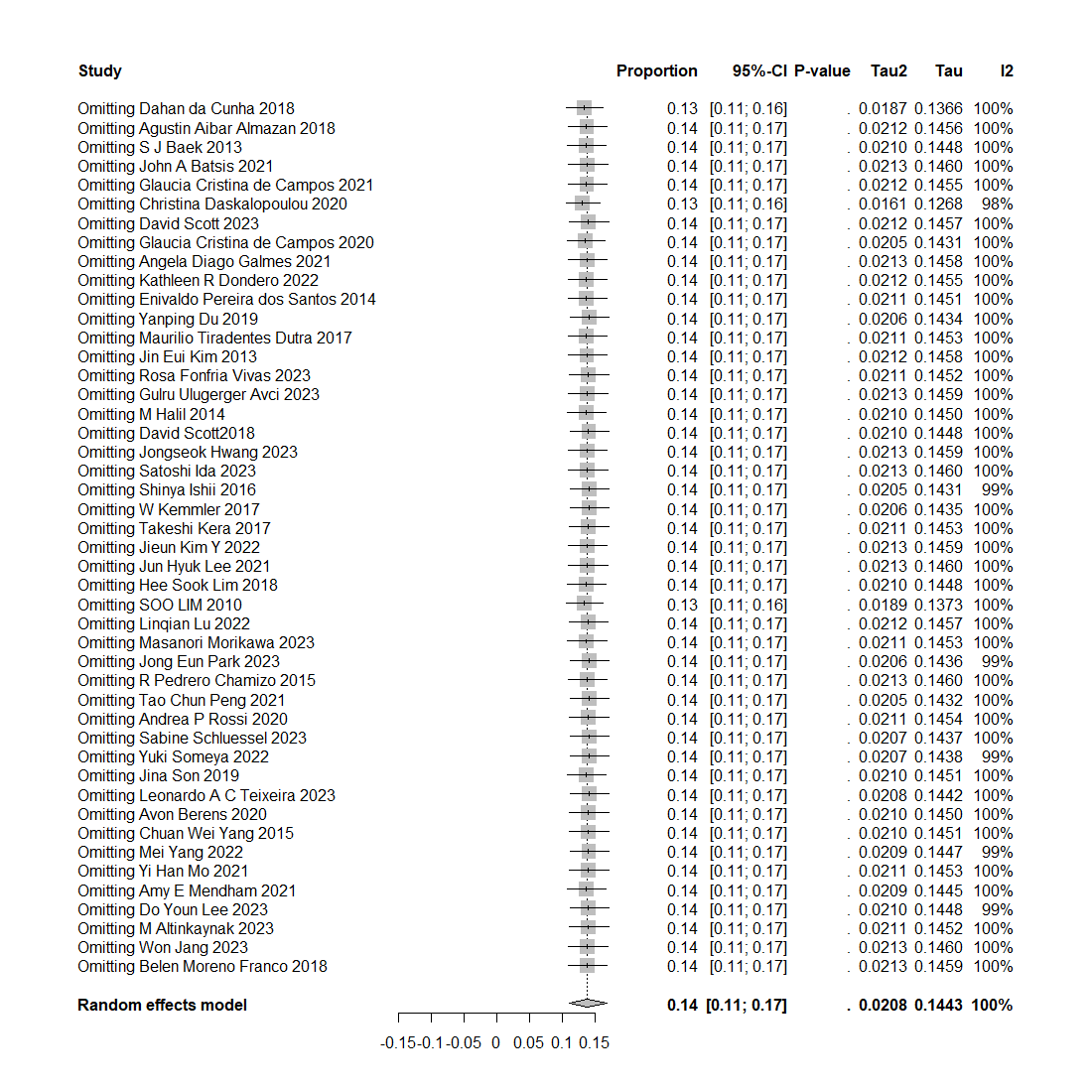

Supplement: Supplementary file 5 — Supplementary Material 5 [file 12877_2024_4952_MOESM5_ESM.docx]
